# Ferulic acid alleviates alveolar epithelial barrier dysfunction in sepsis-induced acute lung injury by activating the Nrf2/HO-1 pathway and inhibiting ferroptosis

**DOI:** 10.1080/13880209.2022.2147549

**Published:** 2022-11-25

**Authors:** Xianming Tang, Jiqiang Liu, Shuo Yao, Jianfei Zheng, Xun Gong, Bing Xiao

**Affiliations:** Department of Emergency Medicine, The Second Xiangya Hospital of Central South University, Emergency and Difficult Diseases Institute of Central South University, Changsha, P. R. China

**Keywords:** MLE-12 cells, cell death, caecal ligation and puncture, ferrostatin-1

## Abstract

**Context:**

Ferulic acid (FA) has antioxidative and anti-inflammatory effects, and is a promising drug to treat sepsis.

**Objective:**

To study the therapeutic effect of FA in sepsis-induced acute lung injury (ALI) and its underlying mechanisms.

**Materials and methods:**

The caecal ligation and puncture (CLP) manoeuvre was applied to establish a murine model of sepsis-induced ALI, and female BALB/c mice (6 mice per group) were subjected to 100 mg/kg FA or 0.8 mg/kg ferrostatin-1 (Fer-1, ferroptosis inhibitor) treatment to clarify the role of FA in preserving alveolar epithelial barrier function and inhibiting ferroptosis. Lipopolysaccharide (LPS; 500 ng/mL)-induced cell models were prepared and subjected to FA (0.1 μM), sh-Nrf2, and Fe (Fe-citrate, ferroptosis inducer; 5 M) treatment to study the *in vitro* effect of FA on LPS-induced alveolar epithelial cell injury and the role of the Nrf2/HO-1 pathway.

**Results:**

We found that FA decreased the lung injury score (48% reduction), lung wet/dry weight ratio (33% reduction), and myeloperoxidase activity (58% reduction) in sepsis-induced ALI. Moreover, FA inhibited ferroptosis of alveolar epithelial cells and improved alveolar epithelial barrier dysfunction. The protective role of FA against alveolar epithelial barrier dysfunction could be reversed by the ferroptosis inducer Fe-citrate, suggesting that FA alleviates alveolar epithelial barrier dysfunction by inhibiting ferroptosis. Mechanistically, we found that FA inhibited ferroptosis of alveolar epithelial cells by activating the Nrf2/HO-1 pathway.

**Conclusion:**

Collectively, our data highlighted the alleviatory role of ferulic acid in sepsis-induced ALI by activating the Nrf2/HO-1 pathway and inhibiting ferroptosis, offering a new basis for sepsis treatment.

## Introduction

Sepsis is a life-threatening syndrome caused by uncontrolled host responses to infection leading to organ dysfunction. The lungs are vulnerable to sepsis-induced injury; it is estimated that more than 40% of patients with sepsis have acute lung injury (ALI), and a large proportion of these patients end up with acute respiratory distress syndrome (ARDS) (Evans et al. 2021). Sepsis (pulmonary or nonpulmonary origin) is the leading risk factor for ARDS, and the mortality of ARDS can be as high as 40% (Bellani et al. 2016). The pathogenesis of ALI/ARDS remains largely elusive, but it has been well characterised that the loss of alveolar epithelial cell integrity and resultant alveolar epithelial barrier dysfunction is an essential driving mechanism in the process of ALI/ARDS (D’Agnillo et al. 2021). Diffuse alveolar damage (DAD) is one of the hallmarks of ARDS and targeting DAD or alveolar epithelial barrier dysfunction is a promising path leading to effective therapy.

Cell death is a critical cause of alveolar epithelial injury, and our recent work revealed that apoptosis of alveolar epithelial cells is a major reason for alveolar epithelial cell loss (Liu et al. 2022). However, ferroptosis, another form of programmed cell death, was recently reported to be of great significance in the process of sepsis and ALI (Zhu et al. 2019; Dong et al. 2021). Specifically, studies offer evidence that ferroptosis also contributes to epithelial barrier dysfunction (Cheng et al. 2021; Ma et al. 2021), yet its role and underlying mechanisms in sepsis-induced ALI need to be further explored.

Ferulic acid (FA) is a natural compound widely found in various kinds of plants and vegetables that can be easily absorbed into the body (Chaudhary et al. 2019). FA is characterised by low toxicity and possesses various physiological potentials, including anti-inflammatory and antimicrobial activities (Zduńska et al. 2018; Li et al. 2021). FA is capable of restraining LPS-induced ALI by alleviating the inflammatory cascade in mice (Mir et al. 2018). Notably, FA can improve intestinal epithelial barrier function by activating the Nrf2/HO-1 pathway (He et al. 2019). As recently highlighted, Nrf2 protects against intestinal ischemia–reperfusion-induced ALI by reducing ferroptosis (Dong et al. 2020). Given this, we propose that FA could inhibit ferroptosis of alveolar epithelial cells and improve alveolar epithelial barrier function through activation of the Nrf2/HO-1 pathway in sepsis-associated ALI.

## Materials and methods

### Ethics statement

All animal experiments were approved by the Ethics Committee of Second Xiangya Hospital, Central South University (Changsha, China) in accordance with the National Institutes of Health Guidelines on the Use of Laboratory Animals (Approval No.: 2020650).

### Animal model

A murine sepsis-associated ALI model was established by the caecal ligation and puncture (CLP) manoeuvre as described previously (Aziz et al. 2018). Briefly, female BALB/c mice (6–8 weeks, weighing 20–25 g, purchased from Hunan SJA Laboratory Animal Co., Ltd., Changsha, Hunan, China) were raised in specific pathogen-free conditions under controlled temperature (23–25 °C) and humidity (40–80%) as well as a 12 h dark/light cycle for 1 week of acclimation. They were fed a standard chow diet and water *ad libitum*. Mice were anaesthetised with 2% isoflurane inhalation and underwent moderate caecal ligation and puncture in accordance with a previously reported protocol (Rittirsch et al. 2009). Meanwhile, mice in the control groups were subjected to a sham operation. Buprenorphin (0.05 mg/kg) was injected for postoperative analgesia, and all mice were placed in cages immediately after the surgical procedures with free access to water and food (Rittirsch et al. 2009). All mice were randomised into (6 mice per group) the control group, CLP group, CLP + FA group (CLP mice that were administered 100 mg/kg FA), Fer-1 group (sham operation mice that were administered the ferroptosis inhibitor ferrostatin-1, 0.8 mg/kg), and CLP + Fer-1 group (CLP mice that were administered Fer-1, 0.8 mg/kg). FA and Fer-1 were first dissolved in DMSO to prepare stock solutions, and aliquots of the stock solutions were then diluted to the final concentration with PBS solution (Liu et al. 2020; Wu et al. 2021). All drug interventions were injected intraperitoneally 1 h prior to the CLP manoeuvre. Subdermal normal saline injection (1 mL) was administered to each mouse for the purpose of fluid resuscitation. Sixteen hours post-operation, the mice were euthanized, and bronchoalveolar lavage fluid (BALF) and lung tissues were collected for the subsequent experiments. BALF was collected, and BALF protein levels were measured in accordance with methods used in our previous study (Liu et al. 2022).

### Histopathological examination

Right lungs were inflated with 20 cm H_2_O pressure to maintain near-physiological architecture (Matute-Bello et al. 2011), and then specimens were fixed in 4% paraformaldehyde. Fixed specimens were embedded with paraffin, sectioned into slices of 4 μm in thickness, deparaffinized with xylene, and stained with haematoxylin and eosin. A semiquantitative scoring system in accordance with the American Thoracic Society workshop report was applied to evaluate lung injury (Matute-Bello et al. 2011; Liu et al. 2022).

### Lung wet/dry (W/D) weight ratio

Lung edoema was assessed using the wet/dry weight ratio (Liu et al. 2022). Briefly, the upper lobe of the left lung was separated and weighed (wet weight, W). Afterward, the lung tissues were stored at 80 °C for 24 h and then weighed (dry weight, D). The W/D ratio equals the wet weight divided by the dry weight.

### Measurement of myeloperoxidase (MPO) activity

MPO activity was measured in lung tissues as an index of neutrophil accumulation and lung inflammation as previously reported (Zhou et al. 2019). Briefly, lung tissues were ground into homogenates and centrifuged, and the supernatants were collected. Measurement of MPO activity was implemented utilising the MPO colorimetric activity assay kit (Nanjing Jiancheng Co., Ltd., China) in accordance with the manufacturer’s protocol.

### Measurement of iron level, malondialdehyde (MDA) content, glutathione (GSH) level, and reactive oxygen species (ROS) level

Endogenous total, ferric, and ferrous levels of lung tissues were determined with an iron assay kit (Abcam, USA) as described previously (Qiang et al. 2020). Briefly, lung tissues were washed with cold PBS buffer and then homogenised. After centrifugation, the supernatants were collected for an iron assay in accordance with the manufacturer’s protocol. MDA was measured with commercial MDA assay kits (Nanjing Jiancheng, China) as previously described (Qiang et al. 2020). Lung tissues were ground into homogenates and centrifuged, and the supernatants were used for further MDA content detection according to the manufacturer’s protocol. GSH levels were determined with a standard GSH assay kit (Nanjing Jiancheng, China) in accordance with a previous report and the manufacturer’s protocol (Qiang et al. 2020; Chen et al. 2021). Intracellular ROS levels were determined with a fluorescence probe, 2′,7′-dichlorodihydrofluorescein diacetate (Invitrogen, CA), in accordance with a previous report and the manufacturer’s protocol (He et al. 2019).

### Cell culture and transfection

MLE-12 cells were cultured with DMEM/F12 medium supplemented with 10% foetal bovine serum in addition to 1% penicillin and streptomycin in a 5% CO_2_ incubator at 37 °C. Nrf2 knockdown was achieved by transfection of shRNA. Once cultured MLE-12 cells reached 70% confluence, equal volumes of Lipofectamine 2000 (Invitrogen, Carlsbad, CA), and sh-NC or sh-Nrf2 were added for 6 h. After removal of transfection solutions, the cells were cultured in baseline medium for another 12 h before they were further treated. The shRNA sequence used was shNrf2: CCG GGC TCC TAC TGT GAT GTG AAA TCT CGA GAT TTC ACA TCA CAG TAG GAG CTT TTT. LPS (*Escherichia coli* LPS O55:B5; Sigma–Aldrich, St. Louis, MO) was first dissolved in a stock solution (5 mg/mL), diluted to a final concentration of 500 ng/mL, and treated for 24 h (Liu et al. 2022). In certain groups, cells were treated with FA (0.1 μM) or Fe (ferroptotic inducer, Fe-citrate, 5 M, Selleck Chemicals, Houston, TX) (Wang et al. 2022) before LPS stimulation. Cells without LPS stimulation were considered the control group. For LPS intervention experiments, the foetal bovine serum concentration was adjusted to 1%. All experiments were repeated in triplicate.

### Cell viability detection

Cell viability was evaluated with a Cell Counting Kit-8 (CCK-8, Dojindo, Tokyo, Japan) assay in accordance with a previous report (He et al. 2019) and the manufacturer’s protocol. Briefly, cells were plated in 96-well plates at a density of 5000 cells per plate. After treatments, 10 µL of CCK-8 solution was added to each well and then incubated for 3 h. The optical density (OD) values were measured at 450 nm using an MRX II microplate reader (Dynex, Chantilly, VA).

### Quantitative real-time polymerase chain reaction (qPCR)

qPCR experiments were carried out as previously described (Liu et al. 2022). In brief, total RNA extraction was implemented employing the TRIzol^®^ kit (Thermo Fisher Scientific). The cDNA Synthesis kit (Takara, Otsu, Shiga, Japan) was adopted to synthesise cDNA. qPCR was implemented utilising SYBR-Green Supermix (Invitrogen Life Technologies). The relative expression level was calculated employing the 2^−ΔΔ^*^Ct^* method with GAPDH as a normaliser. The primers used are summarised in Table 1.

**Table 1.** Primer sequences.

	Sequences
Nrf2	F: 5′-TTCTTTCAGCAGCATCCTCTCCAC-3′
	R: 5′-ACAGCCTTCAATAGTCCCGTCCAG-3′
HO-1	F: 5′-CCTCACTGGCAGGAAATCATC-3′
	R: 5′-CCTCGTGGAGACGCTTTACATA-3′
ZO-1	F: 5′-CACCACAGACATCCAACCAG-3′
	R: 5′-CACCAACCACTCTCCCTTGT-3′
Occludin	F: 5′-TCTCAGCCGGCATACTCTTT-3′
	R: 5′-ATAGGCTCTGTCCCAAGCAA-3′
Claudin-1	F: 5′-TTCTCGCCTTCCTGGGATG-3′
	R: 5′-CTTGAACGATTCTATTGCCATACC-3′
GPX4	F: 5′-CCTCTGCTGCAAGAGCCTCCC-3′
	R: 5′-CTTATCCAGGCAGACCATGTGC-3′

### Western blotting assay

The protein expression of ZO-1, occludin, claudin-1, Nrf2, HO-1, GPX4, and GAPDH was determined by western blotting as previously described (Liu et al. 2022). Briefly, lung tissues were lysed with RIPA lysis buffer (Beyotime, China) and then subjected to electrophoresis separation. The separated protein was transferred onto PVDF membranes, which were blocked with 5% non-fat milk for 1 h and incubated with primary antibodies (Nrf2, ab92946; HO-1, ab13243; GPX4, ab231174; ZO-1, ab96587; occludin, ab222691; claudin-1, ab180158; GAPDH, ab22555) at 4 °C overnight. Then, the membranes were incubated with secondary antibody for 1 h (Santa Cruz Biotechnology, Santa Cruz, CA) before they were washed three times with TBST buffer. After washing, the protein bands were visualised by ECL reagent and then quantified by a Gel-Pro analyser. The results were expressed as the ratio of target protein versus GAPDH.

### Cell permeability assay

Measurement of cell permeability was carried out using transepithelial electrical resistance (TEER) and FITC dextran flux as previously described (He et al. 2019). TEER was tested with a voltohmmeter (MillicellERS-2, Millipore). Dextran flux was measured with FITC-dextran (Sigma). FITC intensity was tested utilising a microplate reader (E8051, Promega).

### Statistical analysis

All quantitative data are presented as the mean ± standard deviation (SD), and comparisons among multiple groups were analysed by one-way ANOVA followed by Tukey’s *post hoc* test. Data were analysed with SPSS software version 22.0. Differences were considered statistically significant when *p* < 0.05.

## Results

### FA improves alveolar epithelial barrier function in sepsis-induced ALI

**Figure 1.**
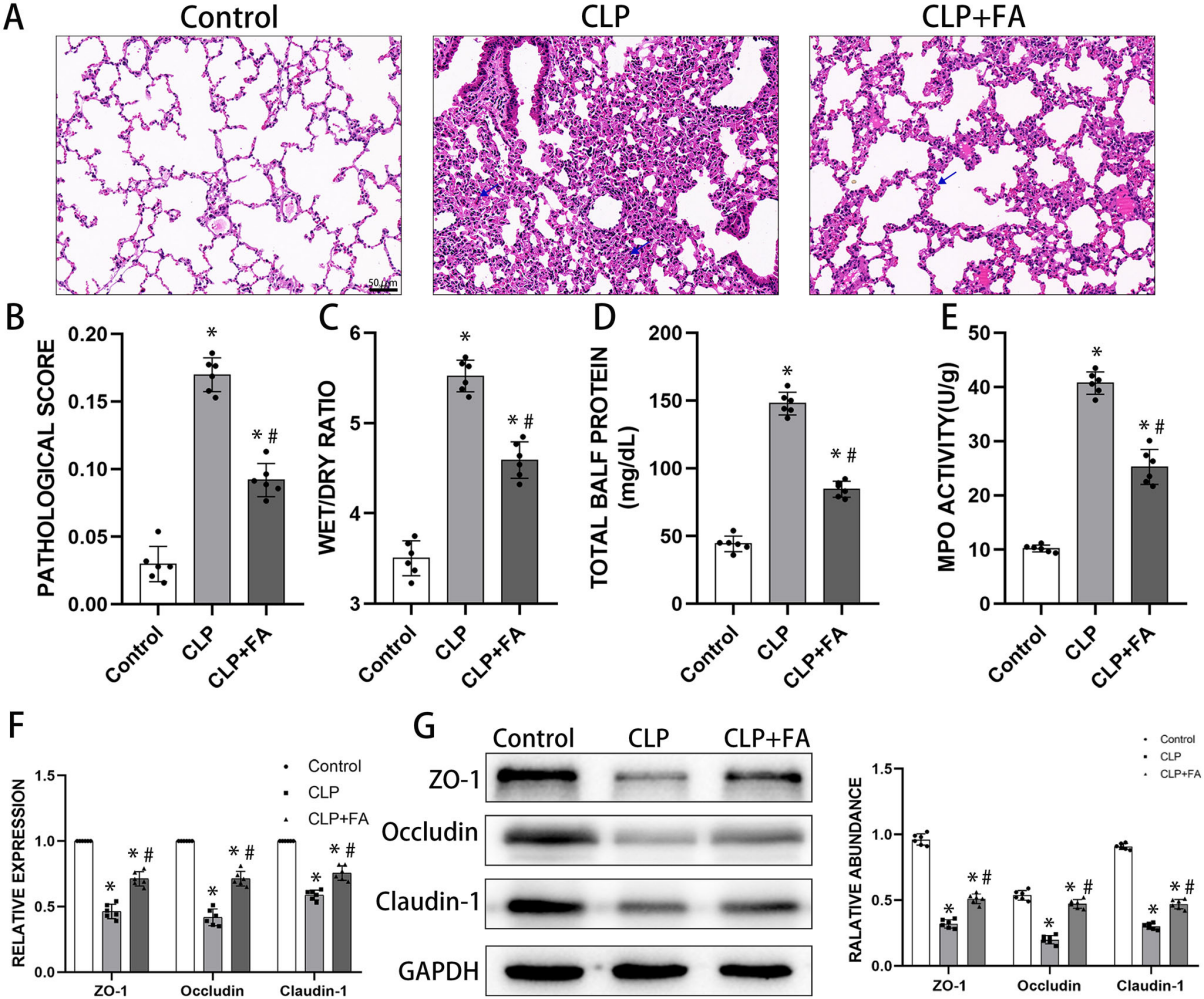
FA affects alveolar epithelial barrier function in sepsis-induced ALI. (A) Representative lung Section H&E staining images of mice that received sham operation or CLP. (B) Pathological score statistics of the three experimental groups. (C) Lung wet-to-dry ratios. (D) Total BALF protein concentrations measured by BCA assay. (E) MPO activity in lung tissues. (F) mRNA expression of tight junction proteins. (G) Protein expression of ZO-1, occludin, and claudin-1 in the lungs measured by western blotting. GAPDH served as an internal control. Original magnification 200×. *p < 0.05 (compared to the control group). ^#^*p* < 0.05 (compared to the CLP group).

First, we established a murine model of sepsis-induced ALI using a moderate caecal ligation and puncture manoeuvre (Rittirsch et al. 2009). Sixteen hours after the operation, lung tissues showed severe structural abnormalities, alveolar atrophy and inflammatory cell infiltration (as indicated by the blue arrow). These pathological abnormalities were markedly alleviated by FA treatment (Figure 1(A)). The lung injury scores paralleled the pathological observations (Figure 1(B)). Sepsis-induced ALI and ARDS were characterised by intra-alveolar flooding with protein-rich edoema, leading to an elevated wet-to-dry ratio and BALF protein concentrations. These abnormalities were also ameliorated by FA treatment (Figure 1(C,D)). Neutrophil infiltration into the alveolar and interstitial space is another contributing factor for sepsis-induced ALI, which can be determined by MPO activity in lung tissues. We found that CLP substantially increased MPO activity in the lungs, which was reduced in the FA-treated group (Figure 1(E)). Loss of alveolar epithelial integrity could lead to barrier dysfunction and alveolar edoema, and one kernel mechanism is the impairment of tight junctions (Herrero et al. 2019). After CLP induction, the mRNA and protein levels of the tight junction factors ZO-1, occludin, and claudin-1 in the lungs decreased drastically, indicating alveolar epithelial barrier dysfunction. The reduction in tight junction factors was rescued by FA treatment (Figure 1(F,G)). Taken together, these findings suggested that FA could improve alveolar epithelial barrier function in sepsis-induced ALI.

### FA inhibits ferroptosis in sepsis-induced ALI

**Figure 2.**
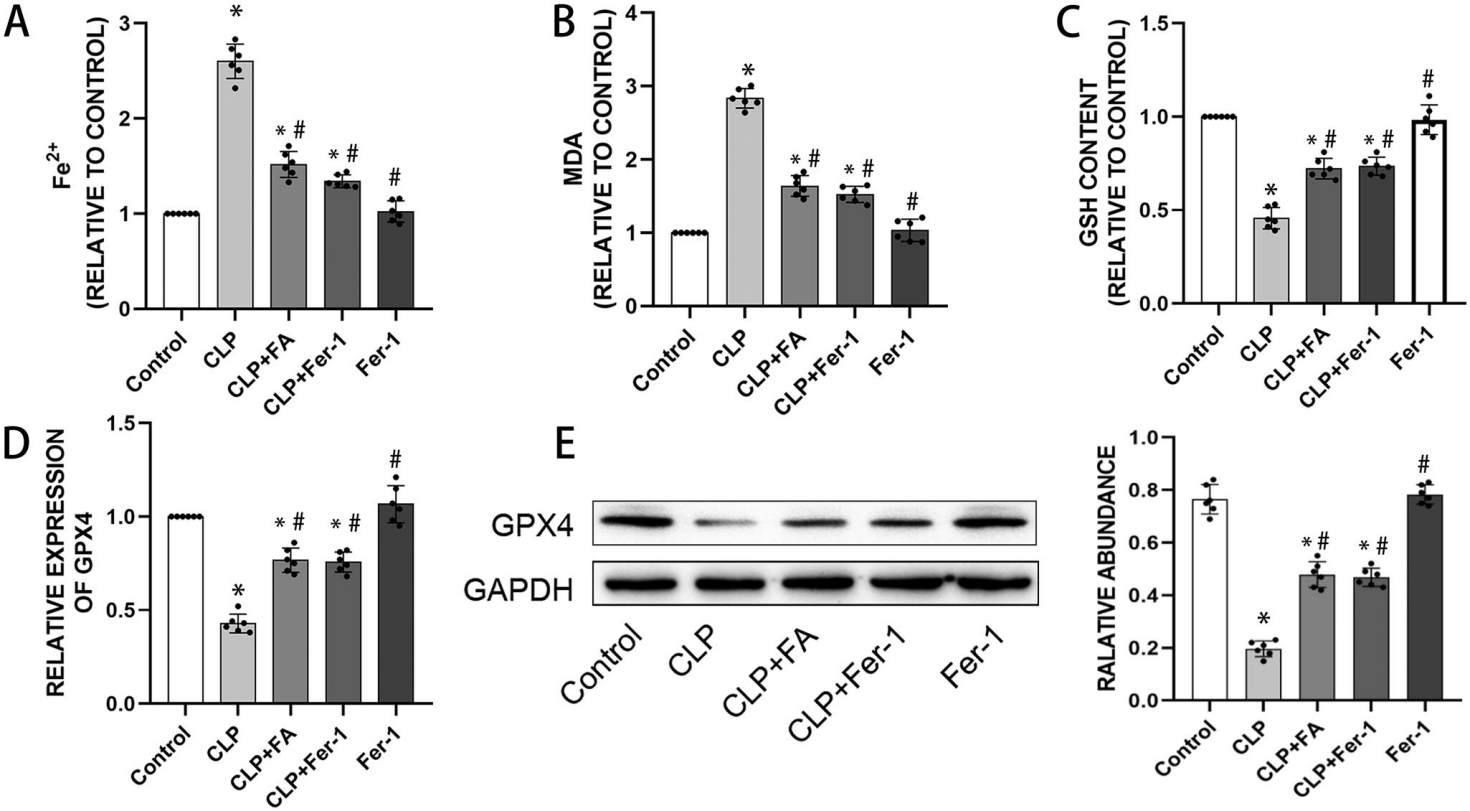
FA inhibits ferroptosis in sepsis-induced ALI. (A) Total iron levels in lung tissues. (B) MDA contents in different experimental groups. (C) GSH levels in lung tissues of different experimental groups. (D) mRNA expression of GPX4 measured with qPCR. (E) GPX4 protein expression was measured by western blotting, and GAPDH served as an internal control. *p < 0.05 (compared to the control group). ^#^*p* < 0.05 (compared to the CLP group). ^$^*p* < 0.05 (compared to the CLP + Fer-1 group).

Now that we have observed impaired alveolar epithelial barrier function in sepsis-induced ALI, which could be reversed by FA, and recent studies have revealed a role of ferroptosis in mediating intestinal epithelial cell barrier dysfunction (Ma et al. 2021), we next explored whether FA exerts its protective role by modulating ferroptosis. We treated CLP-induced mice with FA or the ferroptosis inhibitor Fer-1. Ferroptosis is characterised by the accumulation of iron and iron-dependent cell death (Xu et al. 2021). We first measured the iron levels in lung tissues. Fer-1 treatment did not alter the baseline iron level in lung tissues, while CLP significantly induced iron accumulation. Moreover, FA and Fer-1 treatment both markedly reduced CLP-induced iron accumulation (Figure 2(A)). Elevated MDA levels and decreased GSH content are two other signatures of ferroptosis. We found that CLP increased MDA levels while reducing GSH levels in lung tissues, and these changes were partially yet significantly reversed by FA or Fer-1 treatment (Figure 2(B,C)). Lipid peroxidation is the kernel of ferroptosis, and glutathione peroxidase 4 is a critical inhibitor of lipid peroxidation and a marker of ferroptosis. In CLP-induced mice, GPX4 levels decreased drastically and could be salvaged by FA or Fer-1 treatment (Figure 2(D,E)). Altogether, these results indicated that FA was capable of repressing ferroptosis in sepsis-induced ALI.

### FA activates the Nrf2/HO-1 pathway in sepsis-induced ALI

**Figure 3.**
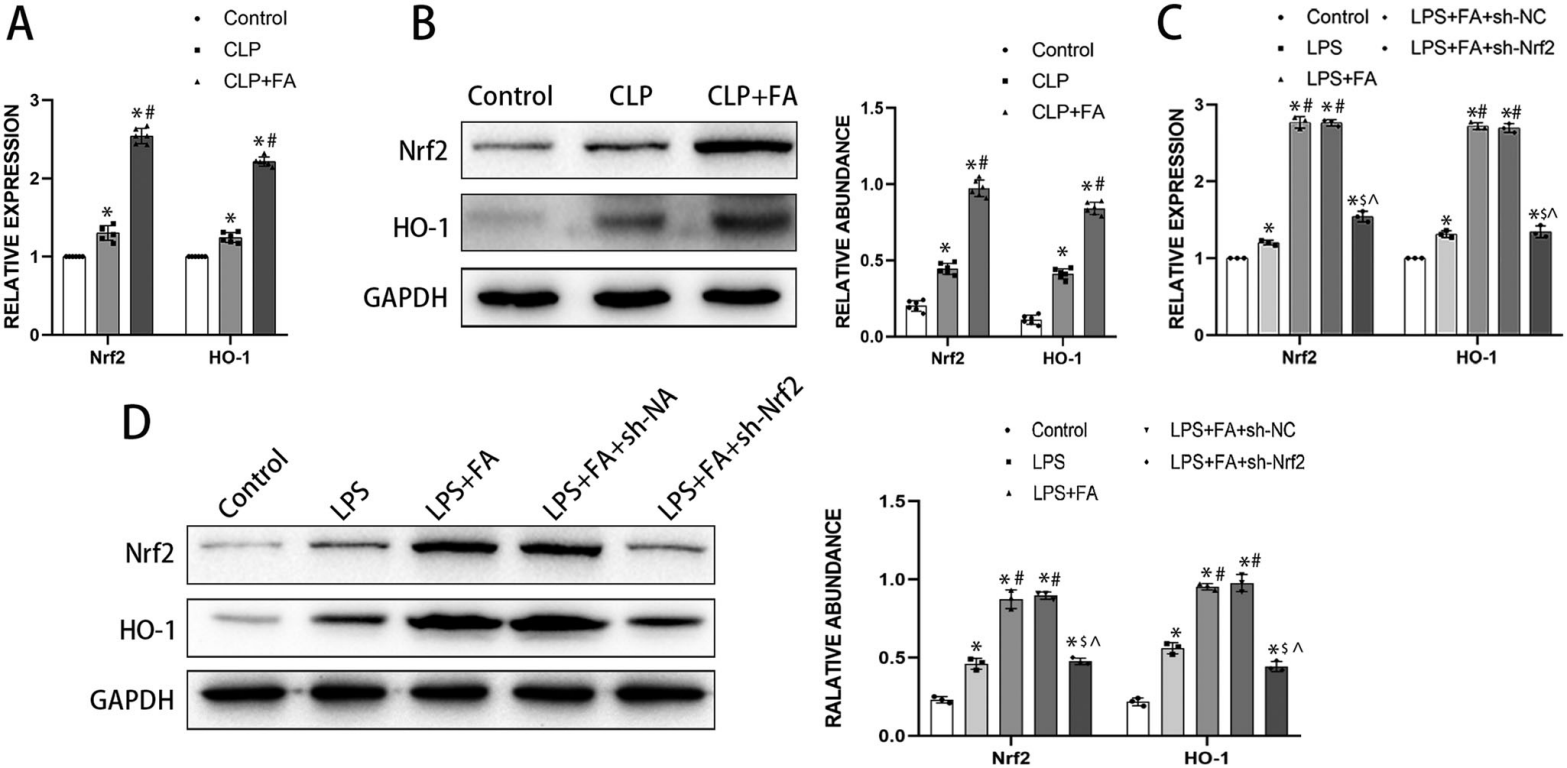
FA activates the Nrf2/HO-1 pathway in sepsis-induced ALI. (A) mRNA expression levels of Nrf2 and HO-1 in the lungs of CLP-treated mice were measured with qPCR. (B) Protein expression levels of Nrf2 and HO-1 in the lungs of CLP-induced mice were measured by western blotting, and GAPDH was used as an internal control. (C) The mRNA expression levels of Nrf2 and HO-1 in LPS-treated MLE-12 cells were measured with qPCR. (D) Protein expression of Nrf2 and HO-1 in LPS-treated MLE-12 cells was measured by western blotting, and GAPDH served as an internal control. *p < 0.05 (compared to the control group). ^#^*p* < 0.05 (compared to the CLP or LPS group). ^$^*p* < 0.05 (compared to the LPS + FA + sh-NC group).

The underlying pathway by which FA regulates sepsis-induced ALI was our next focus. Nrf2/HO-1 signalling is a critical pathway in ferroptosis (Dong et al. 2021; Ryter 2021; Xu et al. 2021), so we postulated that the Nrf2/HO-1 pathway was responsible for the inhibitory role of FA in ferroptosis. As depicted in Figure 3(A,B), CLP induction led to a mild yet significant elevation of Nrf2 and HO-1, implying a compensatory reaction. On the other hand, FA treatment drastically enhanced the expression of Nrf2 and HO-1 at both the mRNA and protein levels. We next treated MLE-12 cells with LPS to establish an *in vitro* assay for sepsis-induced alveolar epithelial cell injury. Our previous study found that 500 ng/mL LPS for 24 h was able to induce significant cell death (Liu et al. 2022). LPS treatment stimulated elevation in Nrf2 and HO-1 expression, while FA treatment further enhanced the transcription and translation of both Nrf2 and HO-1. When MLE-12 cells were transfected with shRNA targeting Nrf2, the expression of both Nrf2 and HO-1 decreased drastically. These data revealed that FA treatment could activate the Nrf2/HO-1 pathway in sepsis-induced ALI.

### FA inhibits ferroptosis in alveolar epithelial cells by modulating the Nrf2/HO-1 pathway

**Figure 4.**
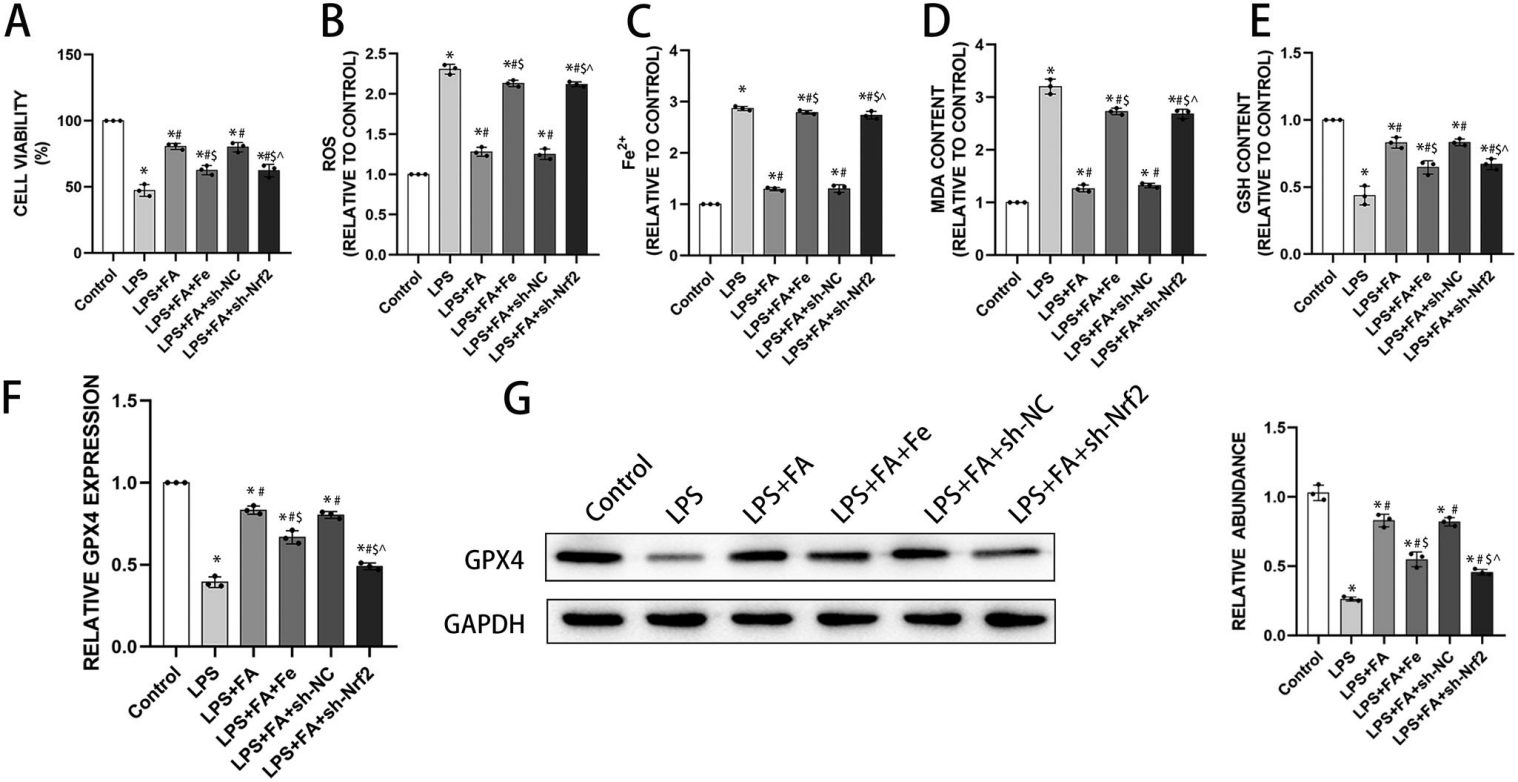
FA inhibits LPS-induced ferroptosis in alveolar epithelial cells by activating the Nrf2/HO-1 pathway. (A) Cell viability was determined with a CCK-8 assay. (B) Assessment of ROS levels in MLE-12 cells treated with LPS (500 ng/mL) for 24 h. (C) Detection of iron levels in MLE-12 cells treated with LPS (500 ng/mL) for 24 h. (D) MDA contents. (E) GSH levels. (F) mRNA expression of GPX4 measured with qPCR. (G) GPX4 protein expression was measured by western blotting, and GAPDH served as an internal control. *p < 0.05 (compared to the control group). ^#^*p* < 0.05 (compared to the LPS group). ^$^*p* < 0.05 (compared to the LPS + FA group). ⁁*p* < 0.05 (compared to the LPS + FA + sh-NC group).

Now that we found that FA can inhibit ferroptosis and modulate the Nrf2/HO-1 pathway in sepsis-induced ALI, we next explored whether FA inhibits ferroptosis *via* the Nrf2/HO-1 pathway. LPS treatment induced significant cell death, which was substantially rescued by FA treatment. Adding iron to the culture media partially reduced the salvaging effect of FA on LPS induction and Nrf2 knockdown (Figure 4(A)). Furthermore, LPS treatment markedly induced intracellular ROS, iron, and MDA accumulation in MLE-12 cells, while GSH content decreased after LPS treatment. FA treatment alleviated these deviations, which were reversed by iron supplementation or Nrf2 knockdown (Figure 4(B,E)). Moreover, LPS intervention reduced GPX4 expression in MLE-12 cells, which was reversed by FA treatment. However, blocking the Nrf2 pathway with shRNA targeting Nrf2 or activating ferroptosis with iron supplementation partially reversed the FA-induced elevation in GPX4 expression (Figure 4(F,G)). In conclusion, the results revealed that FA inhibited LPS-induced ferroptosis in alveolar epithelial cells by activating the Nrf2/HO-1 pathway.

### FA improves alveolar epithelial cell barrier function by inhibiting ferroptosis

**Figure 5.**
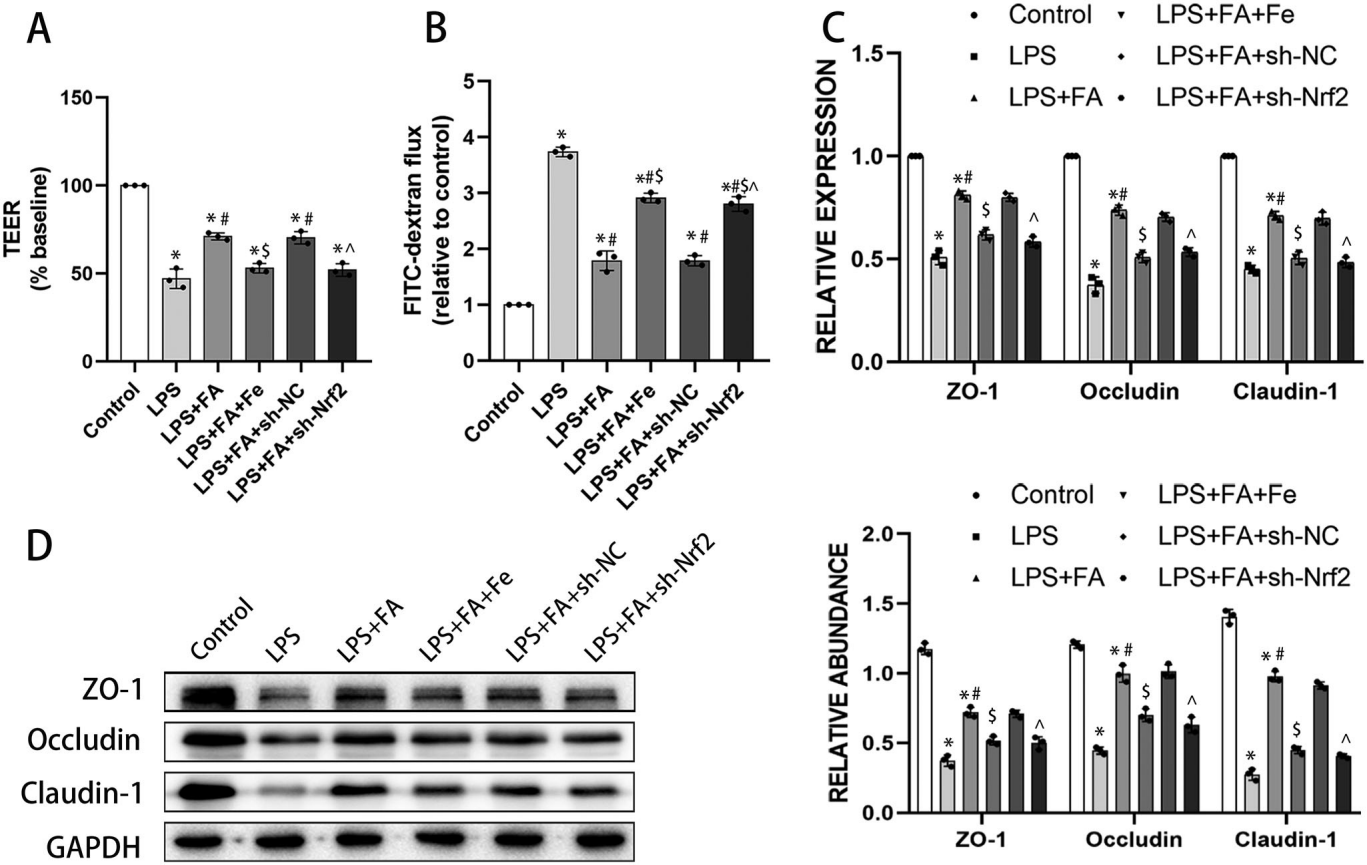
FA improves alveolar epithelial cell barrier function by repressing ferroptosis. Alveolar barrier function was measured with TEER (A) and FITC-dextran flux (B). (C) mRNA expression levels of ZO-1, occludin, and claudin-1 were measured with qPCR. (G) Protein expression levels of ZO-1, occludin, and claudin-1 were measured by western blotting, and GAPDH served as an internal control. * *p* < 0.05 (compared to the control group). ^#^*p* < 0.05 (compared to the LPS group). ^$^*p* < 0.05 (compared to the LPS + FA group). ⁁*p* < 0.05 (compared to the LPS + FA + sh-NC group).

As we have described above, FA can ameliorate sepsis-associated ferroptosis and improve sepsis-induced alveolar epithelial barrier function, and ferroptosis has been reported to contribute to intestinal epithelial barrier dysfunction (Ma et al. 2021). We next explored whether the role of FA in improving alveolar epithelial barrier function is ferroptosis dependent. As expected, LPS treatment impaired barrier function in MLE-12 cells, presented as decreased TEER and increased FITC-dextran flux. FA treatment ameliorated LPS-induced barrier dysfunction, which was abolished by iron supplementation or Nrf2 knockdown (Figure 5(A,B)). Tight junctions are of great importance in maintaining barrier integrity (Otani and Furuse 2020), and LPS treatment decreased expression of tight junction proteins, including ZO-1, occludin, and claudin-1, in MLE-12 cells. FA treatment salvaged the expression of these tight junction proteins in LPS-exposed MLE-12 cells, while those changing tendencies were reversed by sh-Nrf2 or iron supplementation (Figure 5(C,D)). Taken together, these findings indicated that FA improved alveolar epithelial barrier function by repressing ferroptosis.

## Discussion

Organ dysfunction is the inevitable consequence of sepsis, and the lung is one of the most vulnerable organs in sepsis. Currently, the pathophysiology of sepsis-induced ALI remains largely elusive, and effective pharmaceutical therapy is scarce (Meyer et al. 2021). FA is a well-characterised phenolic compound with antioxidative and anti-inflammatory properties that can exert a protective role against sepsis-induced ALI (Bacanli et al. 2014). In the present study, we focussed on the role and mechanism of FA in modulating ferroptosis and alveolar epithelial barrier function. In summary, our data suggested that FA ameliorated ferroptosis-mediated alveolar epithelial barrier dysfunction by activating the Nrf2/HO-1 pathway.

We used the CLP manoeuvre to establish a murine model for sepsis-induced ALI and found that FA improved alveolar epithelial cell barrier function, as evidenced by the recovered expression of the tight junction proteins ZO-1, occludin and claudin-1. Tight junctions (TJs) are well-documented structures that restrain paracellular permeability (Odenwald and Turner 2017; Otani and Furuse 2020). Occludin, claudins, and ZO-1 are constituents of TJs and are capable of maintaining the structure of TJs and epithelial barrier function (Gunzel and Yu 2013; Suzuki 2013). Previous studies have demonstrated that FA is able to facilitate the transcription of ZO-1 and claudin-4 in T84 colonic cells (Bergmann et al. 2009), and pre-treatment with FA protects against heat stress-induced intestinal epithelial barrier dysfunction by elevating TJ expression both *in vitro* and *in vivo* (He et al. 2016). What we have found in this study is consistent with these previous reports, indicating a fundamental role of TJs and alveolar epithelial barrier integrity in the prevention of the pathophysiology of ALI. Our recent study revealed that apoptosis of alveolar epithelial cells contributed to septic ALI (Liu et al. 2022). Herrero et al. (2019) reported that Fas activation, a major mechanism of apoptosis, could lead to altered expression of tight junction proteins and eventually formation of lung edoema in the early stages of ARDS. These studies have revealed a contributing role of alveolar epithelial cell apoptosis in driving ALI progression, yet recently, other forms of cell death were also reported to participate in the pathophysiology of ALI/ARDS, including ferroptosis (Yin et al. 2021).

Ferroptosis is a newly described form of programmed cell death due to iron-dependent excess activation of lipid peroxidation (Yin et al. 2021). Recent studies have suggested that ferroptosis of alveolar epithelial cells augments ALI in a variety of settings. For example, Hui Dong et al. (2021) reported that ferroptosis possesses the potential to induce intestinal ischemia/reperfusion-induced ALI. FA, as a well-recognized antioxidant, inhibits ferroptosis in cardiomyocytes (Liu et al. 2021) and neurons (Gunesch et al. 2020), but its role in modulating alveolar epithelial cell ferroptosis remains unstudied. LPS is one of the major pathogen-associated molecular patterns during sepsis and activates neutrophils, macrophages, dendritic cells, and other cells to trigger oxidative stress (Antonov et al. 2008). Oxidative stress represents a phenomenon regarding the imbalance between the release of ROS and antioxidant defense (Mantzarlis et al. 2017). Damage to the antioxidant system may lead to excessive accumulation of ROS, causing oxidative injury to many macromolecules and important organelles (Avogaro et al. 2008; Zong and Zhang 2017). In particular, oxidative stress is closely correlated with inflammation and may result in sepsis-associated injury (Zolali et al. 2015; Gerin et al. 2016). Consistent with our findings in the present study, FA treatment brought about a reduction in MDA levels and an increase in GSH levels in sepsis-induced rats, highly suggestive of the ameliorative effects of FA in sepsis-induced oxidative damage (Bacanli et al. 2014). Additionally, GPX4 reduces lipid peroxides in cells during ferroptosis (Cao and Dixon 2016). Depletion of GPX4 may trigger the release of ROS and subsequently ferroptosis (Yang et al. 2014). Our data also demonstrated that FA treatment could salvage the LPS- or CLP-induced reduction in GPX4. In summary, in the present study, our data revealed that CLP induction/LPS challenge enhanced ferroptosis, which could be reversed by FA treatment. In addition, FA ameliorated sepsis-induced epithelial barrier dysfunction and ALI by inhibiting alveolar epithelial cell ferroptosis. Taken together, this evidence suggests a universal role of FA in protecting cells from ferroptosis-mediated injury, and more studies are needed to further elucidate this hypothesis.

Furthermore, our results revealed that FA ameliorated ferroptosis-mediated alveolar epithelial barrier dysfunction by activating the Nrf2/HO-1 pathway. A previous study reported that LPS treatment could suppress the expression of the Nrf2/HO-1 axis (Wang et al. 2020), which was consistent with our data. Nrf2 is a critical transcription factor participating in antioxidation processes and ferroptosis (Dodson et al. 2019; Ito et al. 2020). Activation of Nrf2 exerts protective effects against the LPS-induced inflammatory response (Thimmulappa et al. 2006, 2007). Many drugs are being explored to regulate ferroptosis through modulation of Nrf2/HO-1 signalling. For example, gastrodin represses glutamate-induced HT-22 cell ferroptosis through the Nrf2/HO-1 signalling pathway (Jiang et al. 2020). In addition, proanthocyanidin inhibits ferroptosis by activating Nrf2/HO-1 signalling, thus facilitating the recovery of spinal cord injury (Zhou et al. 2020). Partially in line with our data, pre-treatment with FA represses NF-κB-dependent activation of inflammatory pathways and promotes nuclear translocation of Nrf2, leading to elevated Mn-SOD and HO-1 activity, thus overcoming radiation-induced duodenal stress (Das et al. 2017). Our study and previous reports support the stimulatory effect of FA on Nrf2 activation, endorsing its therapeutic use in the treatment of the aforementioned conditions. Further studies, especially well-designed clinical trials, are needed to verify the potential value of FA in a real-world setting.

## Conclusions

**Figure 6.**
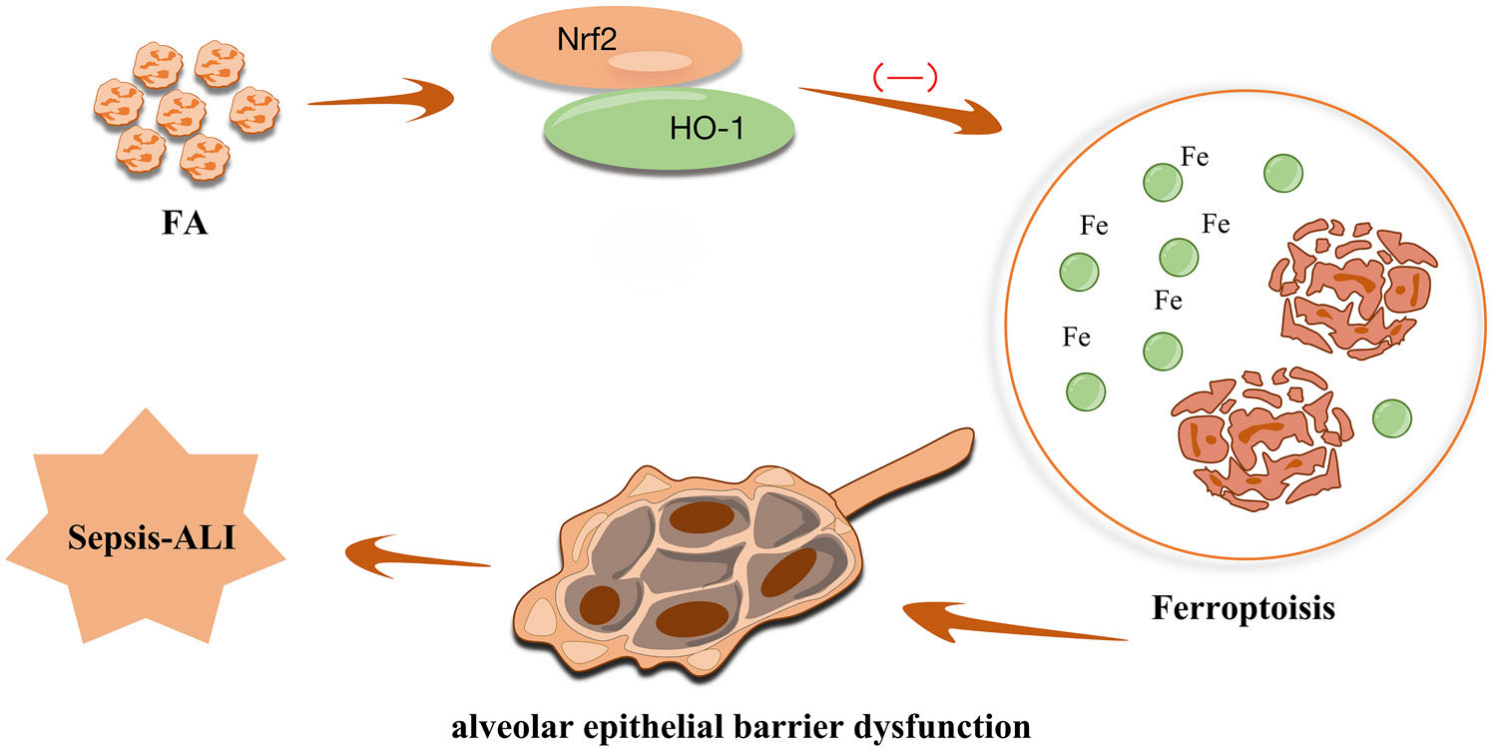
Schematic map of the role of FA in the modulation of sepsis-associated ALI. FA ameliorates ferroptosis-mediated alveolar epithelial cell barrier dysfunction by activating the Nrf2/HO-1 pathway.

The present study found that FA treatment conferred a protective role against sepsis-ALI by inhibiting ferroptosis-mediated alveolar epithelial barrier dysfunction in sepsis-ALI *via* activation of the Nrf2/HO-1 pathway (Figure 6). Our work focussed on the critical role of FA treatment in ferroptosis-mediated alveolar epithelial barrier dysfunction in sepsis-ALI with the involvement of the Nrf2/HO-1 pathway, emphasising a new therapeutic basis for alleviating sepsis-induced ALI.
